# Sulfonated Poly(Ether Ether Ketone)/Praseodymium Doped Zinc Ferrite Composites as Promising Polyelectrolyte Membranes for Fuel Cells

**DOI:** 10.3390/polym17223058

**Published:** 2025-11-18

**Authors:** Laurentiu Baltag, Petrisor Samoila, Corneliu Cojocaru, Mihai Asandulesa, Mariana Cristea, Valeria Harabagiu

**Affiliations:** Romanian Academy, ”Petru Poni” Institute of Macromolecular Chemistry, 41A Aleea Grigore Ghica Voda, 700487 Iasi, Romania; baltag.laurentiu@icmpp.ro (L.B.); samoila.petrisor@icmpp.ro (P.S.); cojocaru.corneliu@icmpp.ro (C.C.); asandulesa.mihai@icmpp.ro (M.A.); mcristea@icmpp.ro (M.C.)

**Keywords:** fuel cells, composite proton exchange membranes, sulfonated poly(ether ether ketone), doped zinc ferrite, solution casting, stability, proton conductivity

## Abstract

Sulfonated poly(ether ether ketone) (SPEEK) is one of the most studied ionic polymers for polymer electrolyte membranes (PEMs) in fuel cells (PEMFCs). To improve its proton conductivity, novel SPEEK/praseodymium-doped zinc spinel ferrite composite membranes of 130–170 μm thickness were prepared via ultrasound-assisted dispersion of various proportions of synthesized doped ferrite nanoparticles into the polymer solution, followed by a simple solution-casting method. The morphology (as observed by SEM and confirmed by DMA) and the conducted physical and chemical tests typical for PEMs, such as water uptake (32–44% at 80 °C), ionic exchange capacity (1.67–1.80 mEq/g), chemical (around 1% loss in Fenton reagent after 24 h), thermal stability (up to 190 °C) and tensile strength (39–50 MPa), were proven to depend on the content of inorganic filler in the composite (up to 5%). The proton conductivity of composite membranes (0.21–2.82 × 10^−2^ S/cm at 80 °C) was assessed by broadband dielectric spectroscopy. The membrane with a content of 0.25 wt.% ZnFe_1.96_Pr_0.04_O_4_ showed the best proton conductivity (3.41 × 10^−2^ S/cm at 60 °C), as compared to 1.60 × 10^−2^ S/cm for Nafion117 measured under the same conditions, demonstrating its suitability as a PEM for fuel cell applications.

## 1. Introduction

To reduce dependence on fossil fuels, alternative renewable energy technologies have been developed. Polymer electrolyte membrane fuel cells (PEMFCs) have attracted significant research and commercial interest due to their mobile and stationary applications. PEMFCs efficiently convert the chemical energy of hydrogen into electrical energy, producing water and heat during operation [1]. Proton exchange membranes (PEMs) are one of the key components that significantly impact fuel cell performances, being essential for the transport of protons from the anode to the cathode while preventing reactant gases (hydrogen and oxygen) crossover. Polymers and composite systems containing acid or basic functional groups that are able to dynamically interact through hydrogen bonding with water or other polar components were considered and tested for their proton-conductive properties. These can be attributed to their unique chemical structure, which is composed of carbon-based hydrophobic backbones and ionic side chain groups. In the presence of water, PEMs exhibit a morphology comprising segregated hydrophobic domains and percolating hydrated ionic channels, through which protons migrate via complex processes [2], mainly through Grotthuss (proton hopping) or vehicle (diffusion) mechanisms.

Perfluorosulfonic acid (PFSA) membranes, such as Nafion, are renowned for their superior long-term stability and high proton conductivity, currently serving as the primary basis for commercially available PEMs. However, the wear from the rather harsh environment during fuel cell running, which leads to both chemical and mechanical degradation, represents the main factor affecting the lifetime of the PEMs [3,4]. The mechanical stress arises from cyclic variations in temperature and humidity, which cause repeated swelling and shrinking during the membrane’s lifetime. Additionally, the presence of water during fuel cell operation can lead to the formation of oxidative byproducts, such as hydroxyl radicals, which accelerate chemical degradation [5,6].

Sulfonated poly(ether ether ketone) (SPEEK) is one of the most well-known and most used poly(aryl ether ketones), considered a good alternative to PFSA membranes due to its advantages such as lower manufacturing cost while maintaining chemical and thermal stability [7,8,9,10,11]. SPEEK is obtained by PEEK sulfonation using concentrated or fuming sulfuric acid or chlorosulfonic acid [12]. SPEEK properties, such as water uptake, physical and chemical stability are highly dependent on the degree of sulfonation [13]. Thus, a recent paper indicated a degree of sulfonation between 51 and 62% as optimum values for SPEEK in PEMFC applications [14].

Different strategies have been developed and used to achieve a better balance between the membrane proton conductivity and its solution, mechanical, and thermal behavior by adding basic groups into the polymer structure [15,16,17] or into the components of the matrix [18,19,20,21,22] by polymer blending [23,24,25,26,27], cross-linking [17,28,29,30] and incorporation of inorganic filler [31,32,33,34]. The cross-linking of the polymer chains improves the resistance to solvents and the dimensional and mechanical stability of the membrane. The cross-linking reactions of the sulfonic acid groups were promoted by high temperature [29,35] or were performed through reactions with alcohols [17,29,36] and other compounds [37,38,39]. The fillers used for composite membranes are various, and they can be graphene [40,41], heteropolyacids [42], silica [43,44,45], metal–organic frameworks [46,47,48] or metal oxides [49,50,51,52,53,54]. In recent years, the research on SPEEK polyelectrolyte membranes usually follows the improvement of the membrane stability or proton conductivity by cross-linking, polymer blending (SPEEK with polysulfones, polyimidazoles, fluorinated polymers, and others [55]), and different new composite strategies [15,33,55,56,57,58]. The most used fillers in recent papers are MOF [47,48], oxides [54,59,60] and ionic liquids (usually used together with MOFs) [31,46,61].

Ferrites, a class of metal oxides, are well known for their thermal stability, magnetic, and catalytic properties. A recent review scrutinized the use of different ferrite structures and ferrite-based composites as catalysts in the composition of electrodes in energy-related processes, especially in energy conversion and storage [62]. However, they were less considered as fillers in polymer electrolyte membranes. To the best of our knowledge, only one report on a polysaccharide/polyurethane blend filled with Ni ferrite was published several years ago [53]. Owing to their relatively hydrophilic nature [63] and the radical scavenging ability [64,65], we investigated the feasibility of SPEEK/ferrite composites for PEMs and examined the influence of filler content on membrane’s physicochemical properties, including proton conductivity. Thus, this paper describes the synthesis of praseodymium-doped zinc ferrite and its use in the preparation of SPEEK-based composite membranes. The effect of the filler concentration on the membrane’s properties was followed by typical tests, such as ionic exchange and water uptake capacities, stability in oxidative environments, and thermal and mechanical behavior. The proton conductivity was evaluated using broadband dielectric spectroscopy.

## 2. Materials and Methods

### 2.1. Materials

Poly(ether ether ketone), PEEK (powder, with an average particle size of 80 microns), concentrated sulfuric acid (98%), metal nitrates (Fe(NO_3_)_3_ × 9H_2_O, Zn(NO_3_)_2_ × 6H_2_O, Pr(NO_3_)_3_ × 6H_2_O), urea (Sigma Aldrich, Steinheim, Germany), and dimethyl sulfoxide (DMSO, Merck, Darmstadt, Germany) were used as received.

### 2.2. Proton Exchange Membranes Preparation

#### 2.2.1. Sulfonation of PEEK

Sulfonated poly(ether ether ketone) (SPEEK) was obtained through direct sulfonation of PEEK, according to a previously reported procedure [66,67]. Briefly, 98% sulfuric acid (40 mL) was poured into a 100 mL Erlenmeyer flask, over which the aromatic polymer (2 g) was added in small portions. The mixture was then kept under continuous stirring at room temperature for one hour, further heated at 50 °C under vigorous stirring for an additional 1.5 h, and then placed in an ultrasonic bath at 60 °C for 15 min. The obtained solution was precipitated in cold distilled water. The resulting polymer beads were washed with distilled water until a constant pH (≈6.3) and dried at 80 °C under vacuum for 24 h.

#### 2.2.2. Synthesis of ZnFe_1.96_Pr_0.04_O_4_

The doped zinc ferrite with the chemical formula ZnFe_1.96_Pr_0.04_O_4_ was synthesized using a sol–gel auto–combustion method [68], modified by using urea as a combustion agent. To obtain 3 g of doped zinc ferrite, solutions of metal salts (a total of 0.0368 mol of metal nitrates at stoichiometric molar ratio (Pr^3+^/Zn^2+^/Fe^3+^ = 0.04/1.00/1.96) in about 60 mL of distilled water and urea (3:1 zinc nitrate/urea molar ratio in about 30 mL distilled water)) were mixed in a Berzelius beaker. The obtained solution was heated up at 80 °C on a water bath, under magnetic stirring, until a viscous gel was formed. The resulting gel was then heated on a sand bath in several steps up to 350 °C to promote the ignition process. The obtained powder was subjected to a thermal treatment at 500 °C for 5 h and at 700 °C for an additional 5 h to ensure the formation of the spinel structure.

#### 2.2.3. SPEEK and Composite Membranes Casting

SPEEK was dissolved in DMSO (concentration, 5% *w*/*w*) at a temperature of 50 °C, under magnetic stirring. For composite membranes, the calculated amounts of filler were first dispersed in a small amount of DMSO using an ultrasonic bath, then added to the polymer solution, and subsequently homogenized under ultrasonic irradiation. The resulting dispersion was poured onto a Petri dish, and the solvent was removed in a vacuum oven at 60 °C for 24 h to obtain easily detachable membranes of 130–170 μm thickness. The composite membranes were designated as SPEEK/Znx, where x represents the weight percentage of the doped zinc ferrite filler added in the membrane (up to 5 wt.%).

### 2.3. Material Characterization

The crystalline structure and the morphology of doped zinc ferrite were followed by wide-angle X-ray diffraction (XRD) on a Shimadzu LabX 6000 diffractometer (Shimadzu Corporation, Kyoto, Japan) and by transmission electron microscopy (TEM) on a Hitachi High-Tech HT7700 microscope (Hitachi, Ltd., Chiyoda, Tokyo) in high-contrast mode at a 100 kV accelerating voltage. The doped ferrite nanoparticles were compacted into a pellet with a hydraulic press and proved to be a hygroscopic material by quick absorption of water drops (see Video S1, recorded with Ossila Contact Angle Goniometer (Ossila, Ltd, Sheffield, UK)). The magnetic properties of the ferrite were observed on a LakeShore 8607 vibrating sample magnetometer (VSM) (Lake Shore Cryotronics, Inc., Westerville, OH, USA). Fourier-transform assisted infrared (FTIR) spectra were registered by using a Bruker Vertex 70 spectrometer (Bruker Corporation, Billerica, MA, USA) on KBr pellets, in the frequency range between 4000 and 400 cm^−1^, with a nominal resolution of 2 cm^−1^ after 32 scans.

The structural characterization of the synthesized SPEEK was performed by proton nuclear magnetic resonance spectrometry (NMR) on a Bruker Avance NEO 400 MHz device (Bruker Corporation, Billerica, MA, USA), in DMSO-d_6_, at room temperature, and standard parameter sets provided by Bruker. The SPEEK sulfonation degree was determined from its ^1^H-NMR spectrum (Figure S1).

The water uptake kinetics of the pristine SPEEK and SPEEK/Znx composite membranes were measured at room temperature and 80 °C. Figure S2 in the Supplementary Material presents the kinetic curves. Membrane samples were dried at 80 °C in a vacuum oven for about 2 h (until constant weight, m_dry_). The dried membrane samples were immersed in 20 mL of distilled water. At regular intervals, up to 4 h, the membrane samples were extracted from the liquid phase, placed between two filter papers to remove the excess water, and weighed (m_t_). The water uptake capacity (WU%) for each point on the kinetic curve was calculated using the formula:

(1) $$WU\left(\%\right)=\frac{m_{t}-m_{dry}}{m_{dry}}\times 100$$

The membrane resistance in the oxidative environment was tested at room temperature. For this purpose, a dry sample (1 cm^2^) was immersed in 15 mL of Fenton reagent (3% H_2_O_2_ and 4 ppm FeSO_4_). The samples were extracted after 24 h, dried, and weighed. The mass loss during immersion was calculated using the initial (m_i_) and final (m_f_) membrane mass:

(2) $$Weight\;loss\left(\%\right)=\frac{m_{i}-m_{f}}{m_{i}}\times 100$$

The ionic exchange capacity (IEC) of the membranes was determined using the acid-base titration method [12]. A membrane was immersed in an acidic solution, 0.01 M HCl, for 24 h to ensure the protonation of all sulfonic acid groups. The membrane was then washed several times with distilled water and a 1:1 ethanol/water solution, dried, weighed, and then immersed in 20 mL 0.01 M NaOH for 24 h. Further, the NaOH solution, in which the membrane was immersed, was titrated with 0.01 M HCl, and IEC was calculated with the following equation:

(3) $$IEC=\left({\left(MV\right)}_{NaOH}-{\left(MV\right)}_{HCl}\right)/M_{p}$$

where M_p_ is the mass of the dried membrane, (MV)_NaOH_ is the molar concentration multiplied by the volume of the sodium hydroxide solution, and (MV)_HCl_ is the molar concentration multiplied by the volume of the hydrochloric acid solution.

The hydration number (λ) at room temperature of SPEEK membranes was calculated with Equation (4) [69]:

(4) $$\lambda =\frac{10\times WU}{18.015\times IEC}$$

The morphology of the membranes was investigated by scanning electron microscopy (SEM) using a Quanta 200 device (Thermo Fisher Scientific, Waltham, MA, USA). The cross-section images of the membranes were obtained on the samples torn apart after being immersed in liquid nitrogen.

The thermal degradation properties of SPEEK and composite membranes were studied in the temperature range of 30–670 °C, with a heating rate of 10 °C/min under a nitrogen atmosphere in Al_2_O_3_ crucibles using a Netzsch STA 449 F1 thermogravimetric analyzer (NETZSCH Analyzing & Testing, Bavaria, Germany).

Dynamic thermo-mechanical experiments (DMAs) were performed on a TA Instruments RSA G2 device (Waters Corporation, Milford, MA, USA) in tension mode. The isochronal experiments (1 Hz) were run with a heating rate of 2 °C/min, from 0 °C up to over 200 °C. The membrane samples with dimensions of 15 mm × 8 mm × 0.25 mm were deformed with a 0.02% strain, which was well within the viscoelastic linear range.

The mechanical properties of the prepared membranes were investigated using a two-column Instron Model 3365 device (Instron Corporation, Norwood, MA, USA) equipped with a 500 N cell force. The uniaxial strain–stress curves were recorded with a displacement rate of 50 mm/min.

To evaluate the proton conductivity of the SPEEK membranes, dielectric measurements were performed using a Novocontrol Concept 40 Broadband Dielectric Spectrometer (Novocontrol Technologies GmbH & Co. KG, Montabaur, Germany) device equipped with an Alpha-A Analyzer and a Quatro Cryosystem temperature controller (Novocontrol Technologies GmbH & Co. KG, Montabaur, Germany). Complex dielectric permittivity of hydrated composite membrane (samples immersed in distilled water for 24 h) spectra were recorded under isothermal conditions by applying an alternating electrical field of 10 mV in a broad range of frequencies from 0.1 Hz to 10^7^ Hz, at temperatures from 20 °C to 100 °C. The polymer films were sandwiched between two gold-plated flat electrodes (provided by Novocontrol), and the measurements were carried out in a pure nitrogen atmosphere. The obtained impedance data sets were evaluated for their validity and consistency using the Kramers–Kronig test (Figure S3 [70]). The proton transport activation energy was calculated with the relation 5 by using the Arrhenius plot:

(5) $$\sigma =\sigma_{0}exp\left(-\frac{E_{\sigma }}{kT}\right)$$

where σ_0_ is a pre-exponential factor, k is the Boltzmann constant, and T is the absolute temperature.

For DMA and proton conductivity measurements, membrane samples with low filler content (under 0.5%) were found to be essential to establish optimal membrane composition.

## 3. Results and Discussions

### 3.1. PEEK Sulfonation

The sulfonation of PEEK preferentially occurs at the phenyl ring between the ether linkages due to the higher electron density of this unit [12]. Starting from a PEEK insoluble sample [12] and following the experimental protocol described in Section 2.2.1, a SPEEK sample with an optimum degree of sulfonation of 61%, as calculated from its ^1^H-NMR spectrum, was prepared. This sulfonation level ensures the appropriate interplay between proton conductivity (increasing with the degree of sulfonation) and hydrodynamic stability of the membrane, which becomes completely soluble in water at temperatures of 80–100 °C, for sulfonation degrees higher than 70–80%.

### 3.2. Doped Zinc Ferrite

A powder of praseodymium-doped zinc ferrite nanoparticles was obtained by the sol–gel auto-combustion technique [68]. As the dimensions of the filler usually influence the properties of composite membranes [71], both doping with praseodymium [72] and urea as the chelating agent (known to ensure a slow combustion process and reduce the dimensions of the resulting particle) [73] were selected in order to allow small ferrite nanoparticles. The structure, morphology and magnetic properties of praseodymium-doped zinc ferrite were investigated with the FTIR, XRD, TEM and VSM methods, respectively.

**Figure 1:**
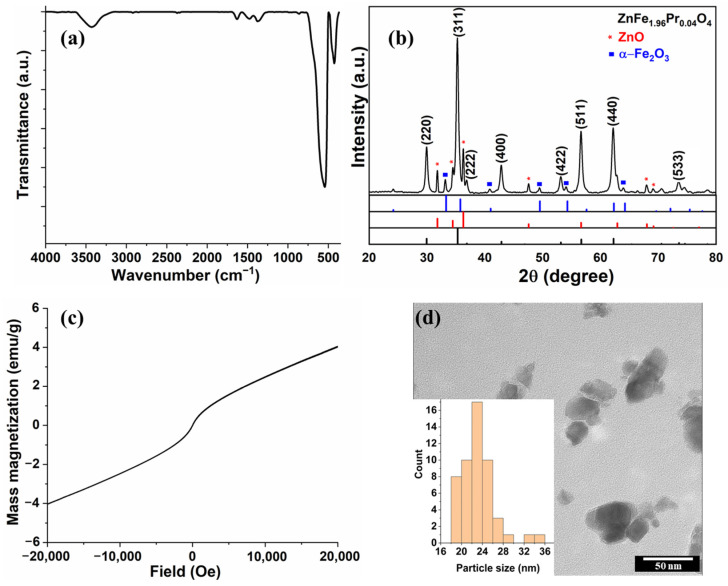
Characterization of ZnFe_1.96_Pr_0.04_O_4_ nanoparticles: (**a**) FT-IR spectra; (**b**) XRD pattern; (**c**) VSM hysteresis curve; (**d**) representative TEM image (insert: particle size histogram).

Figure 1a presents its FTIR spectrum. Two representative bands (427 and 544 cm^−1^) are attributed to the M-O stretching vibration for the cations in the octahedral sites and in the tetrahedral sites [74,75,76], proving the formation of the spinel structure. Absorbed water and possible OH surface groups on the surface of ferrite particles (3400 and 1620 cm^−1^) are also present in the spectrum. Traces of the combustion agent, not fully eliminated by thermal treatment at 700 °C, are still visible in the 1700–1200 cm^−1^ spectral region.

The structure of the spinel ferrite was confirmed by the appearance of the diffraction peaks at 30°, 35°, 37°, 43°, 54°, 57° and 62° in the sample XRD pattern (Figure 1b). The peaks were attributed to the face-centered cubic structure of pure spinel zinc ferrite, as indexed by the JCPDS No. 22–1012 card. Besides the primary phase, one may see in Figure 1b some impurities of α-Fe_2_O_3_ and ZnO, as identified by using JCPDS No. 01-1053 and No. 36-1451 cards, respectively. A simple analysis of the phase ratios revealed a content of 7% for ZnO and 4% for α-Fe_2_O_3_. Other authors attributed the presence of the impurities found in zinc ferrite to the low auto-combustion temperature of urea during synthesis, allowing the aggregation of Fe_2_O_3_ and ZnO phases in the ferrite lattice [73].

The synthesized doped ferrite shows a narrow hysteresis loop shape characteristic of paramagnetic materials (Figure 1c), with a magnetization of 4.03 emu/g at 20 kOe, a remanence of 0.01 emu/g, a coercivity of 0.005 Oe, and an intrinsic coercivity of 13.72 Oe.

The TEM image (Figure 1d) indicates, for the doped zinc ferrite, agglomerated nanoparticles of uneven shapes, with diameters varying between 18 and 36 nm (average diameter of 23 nm) (see histogram in the insert of Figure 1d). Similar results but with larger particle dimensions (average particle size of 48 nm) were recently published for praseodymium-doped zinc ferrite, also obtained by the sol–gel auto-combustion process in the presence of citric acid chelating/combustion agent [77].

The prepared praseodymium doped ferrite proved to be a hygroscopic material (Video S1) and showed lower radical scavenging ability (2,2-diphenyl-1-picrylhydrazyl (DPPH) inhibition of 5%, at 100 μg/mL; see Supplementary Materials) as compared to undoped zinc ferrite (≈26%, 100 μg/mL) [65] and lanthanum-doped zinc ferrite (≈48%, 100 μg/mL) [64]. Due to these properties as well as their low particle size and magnetization value, the doped zinc ferrite nanoparticles were considered as an appropriate filler (without any other modification) to improve the proton conductivity of the SPEEK-based composite membranes.

### 3.3. Composite Membranes

To investigate the influence of inorganic filler content on membrane proton-conducting properties, composite samples containing 0.10, 0.25, 0.50, 1.00, 3.00, and 5.00% doped ferrite nanoparticles dispersed in the SPEEK matrix were prepared according to the procedure described in Section 2.2.3, and their properties were compared with those of the pure SPEEK membrane.

#### 3.3.1. Membrane Morphology

**Figure 2:**
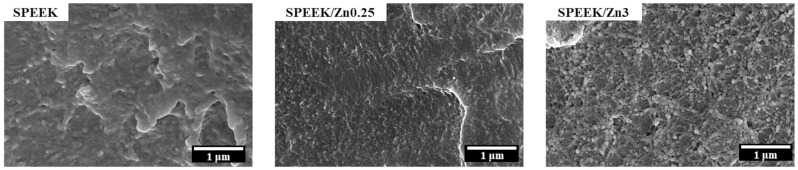
Representative cross-section SEM micrographs of a pristine membrane (SPEEK) and a composite membrane with low filler content (SPEEK/Zn0.25) and high filler content (SPEEK/Zn3).

The morphologies of the prepared membranes were examined by SEM, and representative images are presented in Figure 2. Careful inspection of the micrographs reveals that the membranes exhibit a dense structure without visible pores or cracks, which can be attributed to the slow evaporation of DMSO during the drying process.

Moreover, a uniform distribution of the inorganic filler within the polymer matrix can be visually observed for the SPEEK/Zn0.25 composite membranes, whereas agglomeration of inorganic particles becomes apparent as the filler content increases (SPEEK/Zn3).

#### 3.3.2. Ionic Exchange and Water Uptake Capacities

For proton-conducting membranes, water plays an irreplaceable role, being a key aspect for the performance and functioning of PEMFC [6]. The ionic exchange capacity, the water uptake, and the proton conductivity of the polymeric membranes are directly linked. The connection between IEC, WU, and proton conductivity depends on various factors, including the membrane nature (polymer, filler), thickness, and solvent. Usually, a higher IEC leads to higher WU and better proton conductivity [59,78]. To maintain high proton conductivity, the membrane must maintain a certain level of water, since water is essential for the proton transport through the membrane. Water is transported through PEM by two processes—the diffusion, due to a gradient in water concentration, and the convection, promoted by the difference in pressure at the electrodes [79]. It is necessary for the hydrophilic domains formed by the acid groups to create channels that link one interface to the other. However, above a certain point, the water uptake capacity has a negative impact on the membrane mechanical and dimensional stability, leading to lower performance [21].

As one may see in Figure S2, the water absorption for all SPEEK membranes takes place mostly in the first couple of minutes with minor increase towards equilibrium after 10 min. Table 1 presents the ion exchange capacity at room temperature and the water uptake capacities of the composite membranes at room temperature and 80 °C.

By comparing the characteristic values of pristine SPEEK membrane and those of SPEEK/Znx composite membrane, one may see that the addition of inorganic filler leads to increased IEC and WU at room temperature and a decrease in water uptake capacity at 80 °C, which is more pronounced for SPEEK/Zn3 and SPEEK/Zn5 membranes. The slight increase in WU with higher filler content at room temperature can be attributed to the hygroscopic nature of the doped zinc ferrite (Video S1) and to a stronger interaction between the polymeric matrix and water, as demonstrated by the increase in the hydration number of the sulfonic groups (λ) for membranes with low filler content (0.25 and 1%). The further decrease in λ for membranes of higher ferrite content (3 and 5%) could be explained by poorer interaction with the matrix due to filler agglomeration. By contrast, the water uptake capacity for the composite membranes at 80 °C is lower than that of the pristine SPEEK membrane. For the membrane with a lower content of doped zinc ferrite (0.25 and 1%), the decrease in water uptake capacity is smaller, only about 3–4% compared with the pristine SPEEK membranes. For the membranes with 3% and 5% filler content, the water uptake capacity decreases for both membranes with about 12%.

#### 3.3.3. Oxidative Stability of Membranes

The membrane durability is essential for a long operational lifetime of PEMFC. To assess the suitability of the composite membranes for their usage in PEMFC, the membranes have been tested for their resilience to chemical agents and oxidative species. The resistance to oxidative species was tested using the Fenton reagent. During functioning, radical peroxyl moieties can be produced, and they can attack the membrane surface, reducing the membrane durability [25]. The chemical stability results for the pristine and composite membranes are presented in Table 1. The registered weight loss of the composite membrane is around 1% for all samples. The change in membrane loss does not vary significantly based on membrane composition. These results suggest that the SPEEK/Znx composite membranes are suitable for use in PEMFC.

#### 3.3.4. Thermo-Mechanical Properties

**Figure 3:**
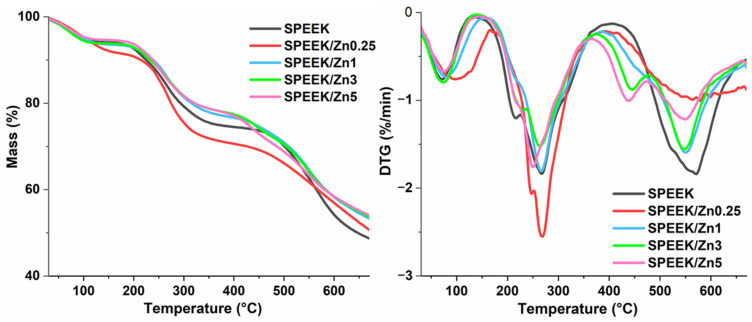
TG and DTG curves of pristine and composite SPEEK membranes.

According to the literature, poly(ether ether ketone) is a thermostable polymer. Its degradation starts at temperatures higher than 550 °C, with a peak at about 570 °C (Figure S4) due to the homolytic cleavage of the polymer chain at the ether bond [80,81]. The initial and major degradation pyrolysis product is phenol. Other volatile products can include dibenzofuran, biphenyl, and naphthalene. The thermal properties of SPEEK membranes were evaluated by TG analysis (Table 2). Figure 3 shows the TG and DTG curves for pristine SPEEK membrane and SPEEK/Znx composite membranes, obtained under a nitrogen atmosphere.

As one may see from Figure 3, the attachment of sulfonic groups on PEEK chains determines the decrease in its thermal stability. Thus, the membrane’s weight loss takes place in three distinct steps. The first observed weight loss with a peak at around 80 °C is due to the dehydration, i.e., the loss of the physically adsorbed water. The second weight loss, which occurs between approximately 200 °C and 340 °C, corresponds to the elimination of the sulfonic acid groups (-SO_3_H) linked to the polymer chains. The third and final step of the membrane weight loss, with a peak at 550 °C, can be attributed to the thermal decomposition of the PEEK polymer backbone. This peak is split into two peaks, less evident for the samples of lower filler contents (SPEEK/Zn0.25 and SPEEK/Zn1), but seen as a more visible shoulder around 450 °C for SPEEK/Zn3 and SPEEK/Zn5 membranes. This shoulder indicates reduced thermal stability at high temperatures, probably due to the possible catalytic effect of the ferrite on the thermal decomposition of the polymer chains or due to the obstruction of polymer packing caused by the more agglomerated ferrite nanoparticles [82], as evidenced by the SEM images (Figure 2). However, the prepared membranes proved appropriate thermal stability for their use in fuel cells based on polymer membranes, which usually work at low and moderate temperatures.

The proton conduction of SPEEK membranes is due to the random distribution of sulfonic groups and the formation of long-range connected hydrated ionic channels, which remain empty following a controlled process of water evaporation, as proved by SAXS [83].

**Figure 4:**
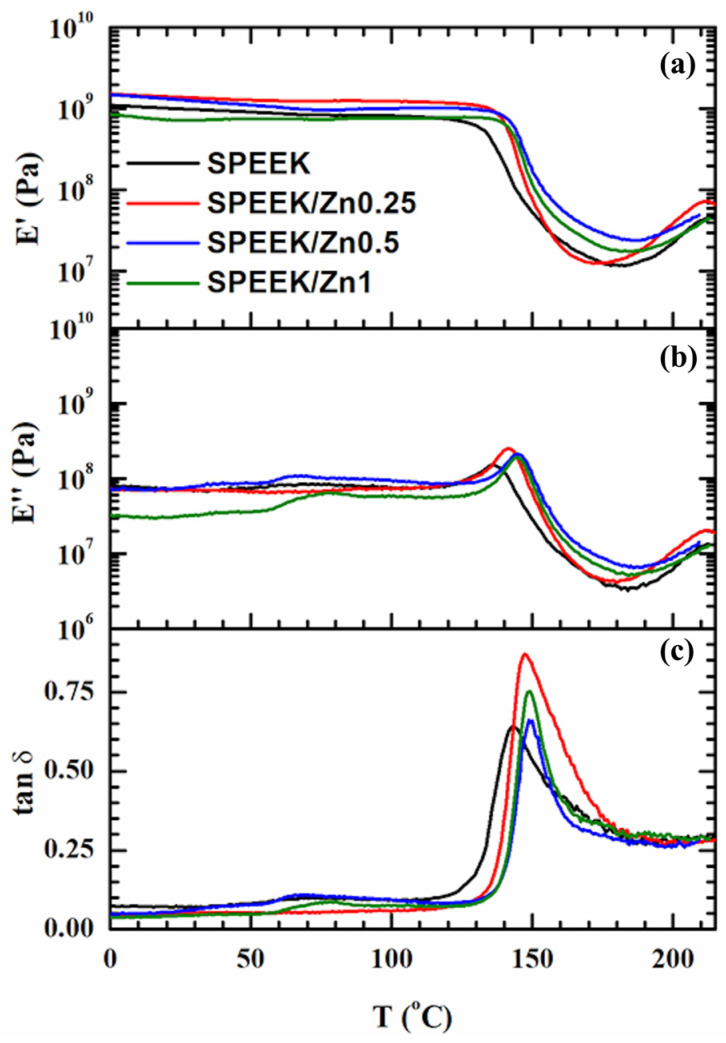
The temperature dependence of the elastic modulus E’ (**a**), loss modulus E” (**b**) and loss factor tan δ (**c**) resulting from the DMA experiment performed at 2 °C/min and 1 Hz for the SPEEK samples.

The dynamic mechanical analysis (DMA) was used to identify the relaxation behavior of the SPEEK membrane (Figure 4), which is an indicator of the molecular mobility. Overall, the viscoelastic behavior of all SPEEK samples follows the same pattern. A β-relaxation is noticed between 50 and 100 °C, which is attributed to the local motions of the backbone [84,85]. The α’-relaxation, associated with the glass transition region in a hydrated state (tg’) (see also the first weight loss in TGA curves, Figure 3), is characterized by a significant drop in the elastic modulus (E’) by about two orders of magnitude (Figure 4a), accompanied by the appearance of peaks in the E” (Figure 4b) and tan δ (Figure 4c) curves at around 150 °C. Typically, a distinct feature of the tan δ peak during the α-relaxation of an amorphous polymer is that the tan δ value at the end of the transition returns to its very small pre-transition level (below 0.1) [86]. In the case of SPEEK samples, the tan δ peaks represented in Figure 4c are asymmetrical. It is evident that the increase in the upward side of the tan δ peak was disturbed and changed to a downward direction. The downward side is less abrupt than the upward one and only goes down to 0.25. It turns out that the main glass transition phenomenon, which supposes long-range coordinated molecular motions, entails another process. This one overlaps the glass transition and induces an increase in stiffness (E’ modulus, Figure 4a) after 175 °C. The gain in polymer rigidity can be attributed to the loss of hydration water, bringing the ionic groups closer and facilitating their aggregation into non-hydrated ionic domains, the real α-relaxation of SPEEK (in the absence of water, i.e., completely non-hydrated state) being around 230 °C [83], which, subsequently, causes the rigidity gain. Hou et al. reported the same process of E’ modulus rise at the end of the glass transition for thermally treated SPEEK samples, which were also obtained in DMSO [35].

All over the investigated temperature range, the elastic component is dominant (E′ > E”). Nevertheless, the E’ modulus vs. temperature profile presents two particularities. One particularity is connected with the values of the E’ modulus of the SPEEK samples in the glassy region. At 25 °C, the order of E’ variation is SPEEK/Zn0.25 (1.4 GPa) > SPEEK/Zn0.5 (1.03 GPa) > SPEEK (1 GPa) > SPEEK/Zn1 (0.723 GPa). This indicates that small quantities of ferrite have a reinforcement effect, while the 1 wt% content leads to a diminishing of the E’ value to below that corresponding to the SPEEK reference sample. This probably happens because of the formation of zinc ferrite aggregates, as the SEM images revealed.

Another particularity refers to the values of the glass transition temperatures (T_g_). Because of complex overlapping phenomena that occur in the second half of the glass transition region, the values of T_g_ associated with E” and tan δ peaks are less reliable. Therefore, the T_g_ values associated with the E’_onset_ are considered for the next discussion: SPEEK (131.1 °C), SPEEK/Zn0.25 (137.8 °C), SPEEK/Zn0.5 (141.1 °C), and SPEEK/Zn1 (140.9 °C). It seems that the presence of doped zinc ferrite in any amount increases the transition temperature, which triggers the long-range coordinated molecular motions attributed to α-relaxation.

The viscoelastic behavior suggests that, among the investigated composites, the sample SPEEK/Zn0.25 has the most homogeneous morphology. The red curve (Figure 4a) displays the steepest fall of E’ during the glass transition region and the more intense increase in E’ after the glass transition. The last one could be characteristic of an easy-going ionic domain aggregation.

**Figure 5:**
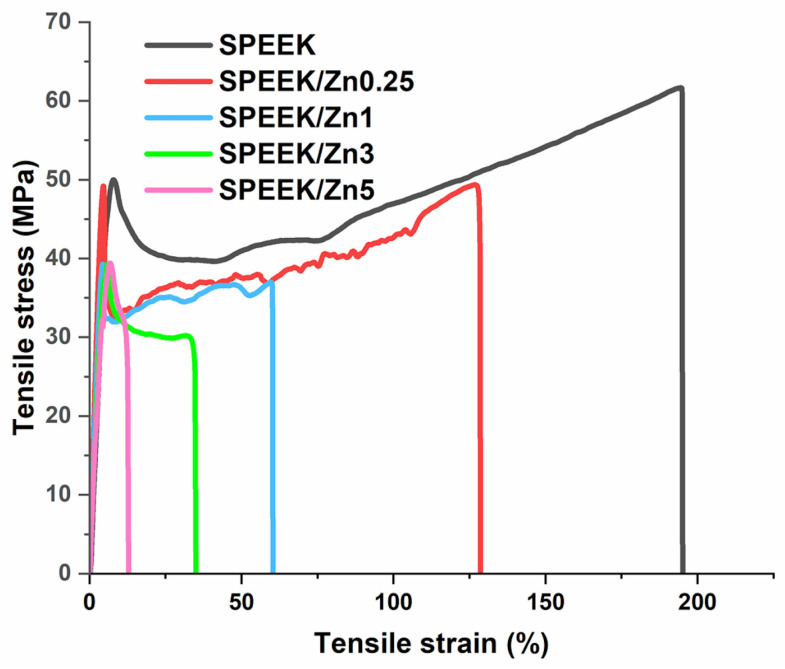
Mechanical stress–strain profiles of pristine SPEEK and SPEEK/ZnFe_1.96_Pr_0.04_O_4_ composite membranes.

To investigate the mechanical behavior of the prepared membranes, stress–strain profiles were recorded at room temperature Figure 5. The determined parameters—tensile strains, tensile stress, and Young’s modulus—at 1% tensile stress are listed in Table 3. All tested membranes presented curves characteristic of plastic materials. At low strain, the samples displayed an elastic region, followed by a yield point and the plastic deformation of the membranes. The introduction of inorganic filler into the SPEEK matrix had a negative impact on the mechanical properties of the composite membranes. As observed in Table 3, a slight decrease in Young’s modulus values and a decrease in tensile strength with increased filler content are registered. The biggest differences between the mechanical properties of pristine SPEEK membranes and SPEEK/Znx composite membranes can be observed for the elongation at break, where a decrease of about one order of magnitude with the increase in the filler content can be seen (195% for the pristine SPEEK membrane to 16% for SPEEK/Zn5 composite membrane). The elastic deformation of the composite membranes does not vary significantly with the increase in inorganic filler; the decrease in elongation at break can be attributed to the hindrance of the polymer chain movement by the inorganic filler.

#### 3.3.5. Broadband Dielectric Spectroscopy

Proton conductivity is perhaps the most important parameter for PEMFC characterization, being directly linked to polymer electrolyte membrane performance. According to the literature, there are two mechanisms for the transport of protons through a hydrated membrane [87]. The “Grotthuss mechanism,” also called hopping mechanism, is considered to have a higher impact. The other mechanism is “vehicle mechanism”, where protons and water (solvent) molecules produce complexes, such as H_3_O^+^, which diffuse through the membrane.

For composite membrane with metal oxides, the inorganic filler can be found both in the hydrophobic polymer backbone and the hydrophilic channels [88], influencing the physicochemical properties of the membranes, such as the membrane microstructure. The metal oxides can interact with water and acid groups (-SO_3_H) through hydrogen bonding, through oxygen and hydroxyl groups present on the nanoparticle surface [2], contributing to WU, IEC and proton conductivity. Qu et al. reported SPEEK composite membranes doped with ammonium ionic liquid and nano-silicon dioxide, observing an increase in WU, IEC and proton conductivity with the addition and increase of SiO_2_, as the silicon dioxide stores water, anchors the ionic liquid and participates in proton conduction with the hydroxyl groups present on the SiO_2_ surface [89].

**Figure 6:**
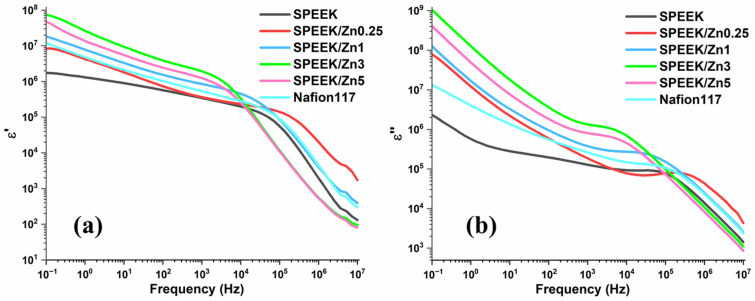
The evolution of dielectric constant (**a**) and dielectric loss (**b**) as a function of frequency at 60 °C for pristine SPEEK, SPEEK/ZnFe_1.96_Pr_0.04_O_4_ composite, and Nafion117 membranes.

To investigate the effect of incorporating ZnFe_1.96_Pr_0.04_O_4_ as a filler in the composite membranes, broadband dielectric spectroscopy measurements were carried out. In Figure 6, the frequency dependences of the dielectric constants (ε’) and of imaginary permittivity (ε”) for the pristine SPEEK, SPEEK/Znx composites and commercial Nafion117 membranes are represented. The decrease in ε’ towards a higher frequency (≥10^5^ Hz, Figure 6a) may be associated with the ionic polarization effect due to the migration of protons through the membrane and dipole orientation.

From Figure 6b, it can be noticed that the dielectric loss of the composite membrane decreases with the increase in frequency. At low frequency, the values obtained for the dielectric loss can be attributed to electrode polarization, which can be observed due to accumulation of free charges at the electrode/membrane interface and Maxwell–Wagner–Sillars (MWS) polarization, which is especially true for the composite membranes with a higher content of filler. At high frequencies (>10^5^ Hz, Figure 6b), the samples showed a steeper slope for dielectric constant and dielectric loss, as the dipoles and mobile charge carriers cannot orient as fast as the applied electric field, leading to a decrease in the parameters. We can examine the Ohmic behavior of the membranes in this frequency range, as the dipoles rarely align with the applied external field at high frequencies, and macroscopic polarization is no longer significant [53]. The shoulder present in the intermediate frequency region can be attributed to the macroscopic polarization of the ionic charges.

The conductivity signal, σ (S/cm), of the composite membranes is connected to the dielectric loss (ε″), the frequency (f), and the vacuum permittivity (ε_0_) and was estimated with the following relation [52]:

(6) $$\sigma =2\pi \varepsilon_{0}f\varepsilon \prime \prime$$

**Figure 7:**
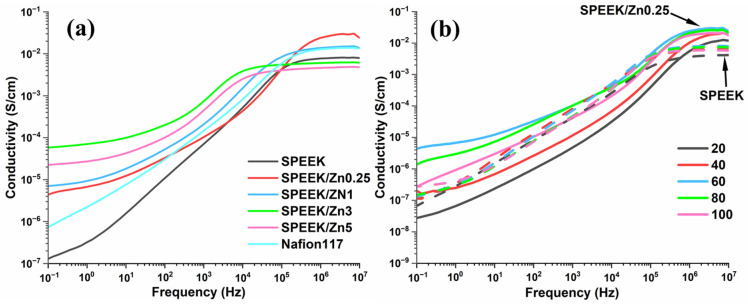
Evolution of conductivity with frequency for (**a**) composite membranes at 60 °C and (**b**) SPEEK (broken lines) and SPEEK/Zn0.25 (full lines) membranes at different temperatures.

Figure 7 displays the conductivity variation as a function frequency at different temperatures for the wet membranes. At all measured temperatures, SPEEK samples show linear dependences on logarithmic frequency, with deviations at high and low frequency (Figure 7a). The frequency-independent conductivity plateau that begins at high frequency (>10^5^ Hz) is mainly due to ionic conductivity mediated by water molecules and sulfonic acid groups. The conductivity changes with the increase in filler content can be observed in Figure 7a. The addition of the inorganic filler in small proportions determines a noticeable increase in the membrane conductivity (SPEEK/Zn0.25 and SPEEK/Zn1) as compared to the pristine membrane (SPEEK), with the optimal content being 0.25 wt%. For the composite membranes with a higher filler content (3 and 5%), the conductivity decreases below the value corresponding to the pristine membrane, probably due to the aggregation of the ferrite, as was observed in SEM images (Figure 2). Similar results were also reported by Sun et al. [90] for SPEEK/WO_3_ composite membranes, where they obtained a higher proton conductivity at low content of WO_3_, attributed to the formation of additional percolated pathways that facilitate the proton transport and a decrease in proton conductivity at high content of WO_3_ due to fewer connecting pathways. The conductivity increases with temperature up to approximately 60 °C, while above this temperature, the proton conductivity is negatively impacted by water loss (Figure 7b).

The activation energy of the composite membranes calculated from the Arrhenius plots (Figure S6) for the 20–50 °C temperature interval (Figure S6) varies from 14.6 kJ/mol for pristine membrane to 19.8, 22.5, 24.8, 25.8, 28.5, and 31.3 kJ/mol for the composite membrane with 0.1, 0.25, 0.5, 1, 3, respectively, of 5% doped zinc ferrite. According to the literature, these values suggest that both “Grotthuss” (proton hopping) and “vehicular” (diffusion) mechanisms are responsible for the proton transport [91,92].

In the Nyquist plot (Figure S5), the Ohmic resistance of the membrane is typically associated with the real-axis intercept of the semicircle at a high frequency. The intercept can be used to obtain the bulk resistance of the membrane. Usually, a line at a low frequency depicts the diffusion process of ions (protons). The proton conductivity (σ (S/cm)) was calculated using the following equation:

(7) $$\sigma =L/(R_{b}\times A)$$

where L is the membrane thickness (cm), R_b_ is the membrane bulk resistance obtained from the high-frequency intercept of the Nyquist plot, and A is the electrode area.

The proton conductivities obtained at different temperatures for the hydrated pristine SPEEK membrane, SPEEK/Znx composite membranes, and Nafion117, used as a reference, are presented in Table 4. As expected, the values for the proton conductivity increase with temperature for all samples up to about 60 °C. Nevertheless, at higher temperatures, the dehydration of the tested membranes leads to a decrease in proton conductivity. Owing the hygroscopic nature of the synthesized doped zinc ferrite, the proton conductivities of SPEEK/Zn0.25 and SPEEK/Zn1 are higher than the conductivity of the pristine membrane. A similar result was also pointed out for SPEEK–zirconia hybrid membranes [60]. Moreover, the presence of ZnFe_1.96_Pr_0.04_O_4_ filler can create additional hydrogen-bonding formations in the composite membrane, a factor that promotes the proton conductivity. The good compatibility between ZnFe_1.96_Pr_0.04_O_4_ and the SPEEK matrix, for SPEEK/Zn0.25 and SPEEK/Zn1 composite membranes, can lead to better phase connectivity; therefore, the formed hydrophilic channels are more favorable to the proton transport through the membrane. However, along with the increase in filler content, a decrease in proton conductivity can be observed for SPEEK/Zn3 and SPEEK/Zn5 composite membranes. This behavior can be linked to the aggregation of ZnFe_1.96_Pr_0.04_O_4_, which hinders the formation of hydrophilic channels and limits the transport of protons. The optimum filler content of the composite membranes is for the SPEEK/Zn0.25 membrane, where the obtained conductivity values are higher than those of SPEEK and Nafion117, under the same experimental conditions, with the best values obtained at 60 °C: 3.41 × 10^−2^ S/cm for SPEEK/Zn0.25 and 1.60 × 10^−2^ S/cm for Nafion117. For the composite membrane SPEEK/Zn1, the results are similar to those of Nafion117 at 60 °C and higher temperatures.

The noticed differences in proton conductivity between Nafion117 and SPEEK/Znx membranes, particularly at elevated temperatures, can be attributed to the strong dependence of the proton transport mechanisms, namely, the Grotthuss and vehicle mechanisms, on the membrane’s water uptake. Under low hydration conditions, proton conduction is predominantly governed by the vehicle mechanism, whereas at higher levels of hydration, the Grotthuss mechanism becomes the dominant pathway for proton transport [2,93].

Table 5 compares the results obtained in this work with the proton conductivity performances of other SPEEK-based composite membranes reported in the literature.

As can be seen from the data in Table 5, the proton conductivity of the SPEEK/Zn0.25 sample has a magnitude similar to the results reported by other authors for membranes containing a SPEEK polymer matrix. Moreover, this sample exhibited a better performance as compared to that of a commercial Nafion117 membrane, measured by broadband dielectric spectroscopy, under the same experimental conditions. However, recent results showed that the introduction of additives with phosphonic groups allows better PEM performances, as a result of their lower energy barrier of phosphonic groups as compared to the sulfonic ones.

## 4. Conclusions

In this study, sulfonated poly(ether ether ketone) (SPEEK) with a degree of sulfonation of 61% was synthesized and used as the matrix for composite membranes. The doped zinc ferrite ZnFe_1.96_Pr_0.04_O_4_ was prepared by the sol–gel auto-combustion method. Composite membranes based on SPEEK containing inorganic filler, with varied weight percentages, have been prepared using a casting method and characterized for their use as a proton exchange membrane in fuel cell. All the composite membranes showed appropriate thermal (up to 190 °C) and chemical stability (maximum 1.2% weight loss in Fenton reagent in 24 h, at room temperature) for use as PEMs. Moreover, the hydrophilic character of the filler conveniently assists the polymeric matrix–water interaction, as IEC and WU measurements evidenced. In the investigated series, the SPEEK/Zn0.25 has the most favorable features, expressed by the even distribution of filler and the highest proton conductivity, i.e., 3.41 × 10^−2^ S/cm at 60 °C, as compared to 1.60 × 10^−2^ S/cm for Nafion117 membrane in the same experimental conditions. These results indicate the usefulness of praseodymium-doped zinc ferrite nanoparticles as fillers to improve the proton conductivity of SPEEK-based membranes, on the one hand, and the appropriateness of the resulting composite membranes as promising candidates for optimization to further enhance their performance as PEMs for fuel cells, on the other hand.

## Figures and Tables

**Table 1:** Membrane physicochemical properties: ionic exchange capacity (IEC), water uptake (WU) capacity (at room temperature and 80 °C), and the weight loss values in Fenton reagent.

Sample	IEC (meq/g)	WU% ^a^ (25 °C)	WU% ^a^ (80 °C)	λ ^b^ (H_2_O/SO_3_H)	Weight Loss (%) ^c^
SPEEK	1.71	24.0	44.2	7.79	0.8
SPEEK/Zn0.25	1.67	24.5	40.2	8.14	1.2
SPEEK/Zn1	1.69	25.3	41.6	8.31	1.0
SPEEK/Zn3	1.78	25.8	32.1	8.05	0.9
SPEEK/Zn5	1.80	26.3	32.6	8.11	0.9

^a^ registered after 4 h. ^b^ λ calculated at room temperature. ^c^ registered after 24 h, at room temperature (Fenton reagent).

**Table 2:** Decomposition temperature of composite membranes.

Sample	Water		Sulfonic Acid Groups			Polymer Backbone			Residual Mass % (at 670 °C)
	T_peak_ (°C)	Mass Loss (%)	T_onset_ (°C)	T_peak_ (°C)	Mass Loss (%)	T_onset_ (°C)	T_peak_ (°C)	Mass Loss (%)	
SPEEK	71	6	190	255	20	464	564	26	49
SPEEK/Zn0.25	100	8	187	267	20	423	558	21	51
SPEEK/Zn1	78	7	197	266	17	433	550	23	53
SPEEK/Zn3	73	6	194	262	16	411	548	22	56
SPEEK/Zn5	75	5	191	250	16	400	550	25	53

**Table 3:** Mechanical characteristics of SPEEK-based membranes.

Samples	Tensile Strength (MPa)	Young’s Modulus * (MPa)	Elongation at Break (%)
SPEEK	49.9	1059	195
SPEEK/Zn0.25	42.9	1130	128
SPEEK/Zn1	39.2	1060	60
SPEEK/Zn3	39.2	1002	34
SPEEK/Zn5	39.4	1001	16

* at 1% tensile stress.

**Table 4:** The proton conductivity calculated from Nyquist plot for the composite membranes.

Samples	Conductivity × 10^−2^ S/cm				
	20 °C	40 °C	60 °C	80 °C	100 °C
SPEEK	0.43	0.64	0.83	0.81	0.63
SPEEK/Zn0.1	0.62	1.14	1.79	1.68	0.71
SPEEK/Zn0.25	1.44	2.75	3.41	2.82	2.25
SPEEK/Zn0.5	0.79	1.41	2.50	2.72	1.80
SPEEK/Zn1	0.50	1.04	1.57	1.26	0.82
SPEEK/Zn3	0.21	0.49	0.62	0.47	0.24
SPEEK/Zn5	0.14	0.29	0.47	0.21	0.02
Nafion117	1.36	1.56	1.60	1.26	0.73

**Table 5:** Comparison of proton conductivities of different SPEEK-based composite membranes.

Membrane	Filler	DS * (%)	Conductivity × 10^−2^ S/cm	RH (%)	Temperature (°C)	Reference
SPEEK	doped zinc ferrite (0.25 wt.%)	61	3.41	-	60	This work
Nafion117	-	-	1.60	-	60	This work
SPEEK	cobalt-based zeolitic imidazolate framework (1 wt.%)	61	0.94	-	80	[21]
SPEEK/SPVdF-HFP (4:1)	sulfated SiO_2_ (6 wt.%)	65	7.90	-	90	[22]
SPEEK/PVDF (9:1)	-	-	0.97	-	80	[25]
SPEEK/PVDF (9:1)	boron phosphate (10 wt.%)	-	3.90	-	80	[25]
SPEEK	sulfated TiO_2_ (0.1 wt.%)	60	6.07	100	-	[59]
SPEEK	SPEEK nanofibers	65	5.58	100	80	[94]
SPEEK/PAI (9:1)	SrTiO_3_ (6 wt.%)	65	1.08	100	150	[95]
SPEEK/PFSA (85:15)	-	68	6.20	-	80	[96]
SPEEK/PFSA (85:15)	Ba_0.9_Sr_0.1_TiO_3_ (6 wt.%)	68	6.70	-	80	[96]
SPEEK/SPI (1:1)	H-MoS_2_ (1 wt.%) and SLS (2 wt.%)	64	5.02	100	80	[97]
SPEEK/PBI (10:1)	-	70	1.70	100	RT	[98]
SPEEK/PBI (10:1)	10% boron nitride	70	2.45	100	RT	[98]
SPEEK	bisphosphonic acid (2 wt. %)	64	22.6	100	60	[99]

* the degree of sulfonation of SPEEK. SPVdF-HFP—sulfonated poly (vinylidene fluoride-co-hexafluoropropylene); PVDF—poly (vinylidene fluoride); PAI—poly (amide imide); PFSA—perfluorosulfonic acid; SPI—sulfonated polyimide; H–MoS2—sulfated molybdenum disulfide; SLS—sodium lignosulfonate; PBI—polybenzimidazole.

## Data Availability

The original contributions presented in this study are included in the article. Further inquiries can be directed to the corresponding author.

## Supplementary Materials

The following supporting information can be downloaded at: https://www.mdpi.com/article/10.3390/polym17223058/s1, Video S1: Hygroscopic properties of doped zinc ferrite; Figure S1: ^1^H-NMR spectra of SPEEK; Figure S2: Water uptake kinetics at room temperature and at 80 °C; Figure S3: The Kramers–Kroning test for the data set of SPEEK membrane at 20 °C; Figure S4: TG/DTG curves of PEEK; Figure S5: Nyquist plot for the SPEEK/Zn0.25 membrane at different temperatures; Figure S6: The Arrhenius plot of conductivity as a function of temperature for SPEEK and SPEEK/doped ferrite composite membranes.
