# Supplementary material for: Sulfonated Poly(Ether Ether Ketone)/Praseodymium Doped Zinc Ferrite Composites as Promising Polyelectrolyte Membranes for Fuel Cells
Source: Polymers (Basel). 2025 Nov 18;17(22):3058. doi: 10.3390/polym17223058 (PMC12656526; doi:10.3390/polym17223058)
Supplement: Supplementary file 1 [file polymers-17-03058-s001.zip › polymers-3930179-supplementary.pdf]

Sulfonated Poly(Ether Ether Ketone)/Praseodymium Doped Zinc Ferrite Composites as Promising Polyelectrolyte Membranes for Fuel Cells

Laurentiu Baltag [1], Petrisor Samoila [1], Corneliu Cojocaru [1], Mihai Asandulesa [1], Mariana Cristea [1], Valeria Harabagiu [1,*]

[1] Romanian Academy, "Petru Poni" Institute of Macromolecular Chemistry, 41A Aleea Grigore Ghica Voda, 700487 Iasi, Romania
*Correspondence: hvaleria@icmpp.ro; Tel.: 0040-741027090

Supplementary Materials

Determination of the degree of sulfonation from ^{1}H-NMR

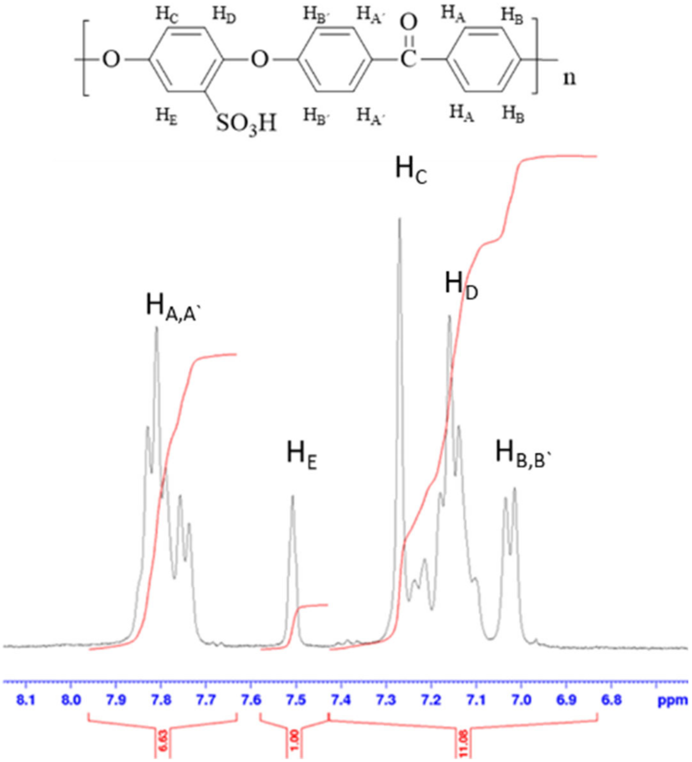

Figure S1. ^{1}H-NMR spectra of SPEEK

The sulfonation product of PEEK with sulfuric acid was recorded in deuterated dimethyl sulfoxide (DMSO-d_6) and is presented in Figure S1. It should be noted that the ^{1}H-NMR proton signal from the hydrogen present in the acid group (-SO_3H) is usually difficult to record due to its labile nature. The sulfonation of PEEK can only take place, in normal conditions, at the phenyl ring between the ether linkages. As the presence of the (-SO_3H) functional group shifts the signal

of the neighboring H_E proton, compared with the other protons in the hydroquinone ring, this proton is being presented as a singlet at approximately 7.5 ppm. For the calculation of the polymer degree of sulfonation (DS), if we consider a polymer with a 100% degree of sulfonation, then the ratio between the integral of the signal area of hydrogen atoms in the ortho position with sulfonic groups and the sum of the integrals of the areas of the other hydrogen atoms on the benzene nuclei is 1/10. For PEEK, the above ratio should be 0/12. The degree of sulfonation can be calculated using equations 1 and 2, where we can state that the ratio of the discussed integral areas is equal to n/(12 - 2n). In the equation, n can take values between 0 and 1 (unmodified and fully sulfonated), 12 represents the number of hydrogen atoms in an unsubstituted monomer unit, and 2n comes from the difference in the number of hydrogen atoms appearing in ^1H NMR, excluding the hydrogen atoms in the ortho position (H_E) of the sulfonic group ($-SO_3H$).

$$\frac{n}{(12-2n)} = \frac{A_{H_E}}{\sum A_{H_{A,A',B,B',C,D}}} \quad \textbf{(1)}$$

Degree of sulfonation (DS) = n * 100 (%) **(2)**

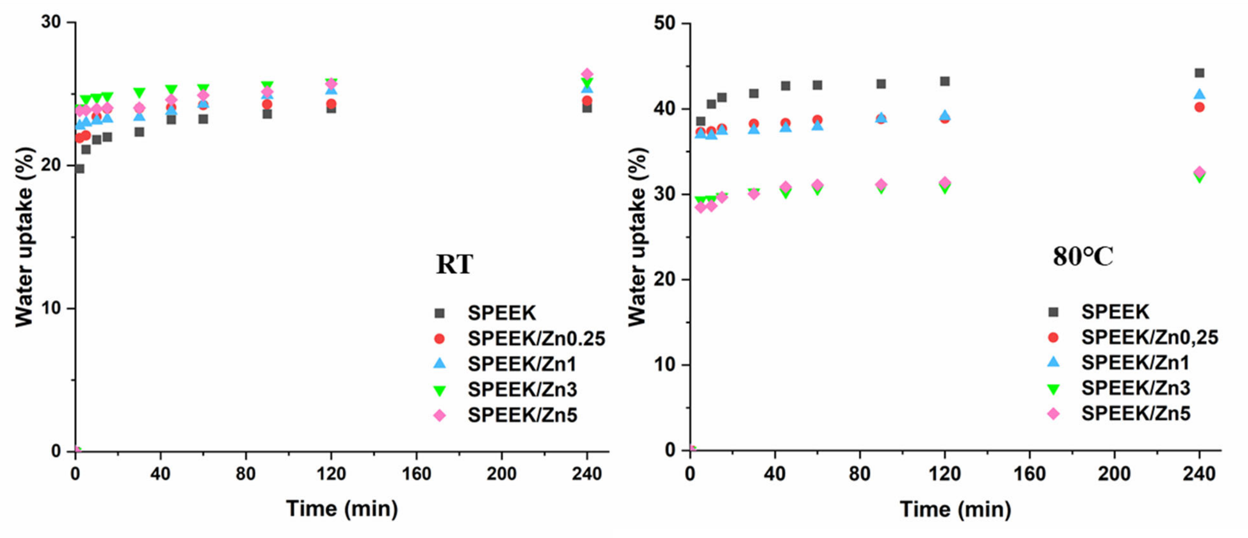

Figure S2. Water uptake kinetics at room temperature and at 80°C.

Kramers-Kroning test

The Kramers-Kroning test was performed using AfterMath version 1.6.10523 [70]. The program fits the experimental data using an equivalent circuit that satisfies the Kramers–Kronig

relations; therefore, any data set that can be successfully fitted can be considered to pass the test. The values for the statistic coefficient, χ^2, were lower than 0.006 for all the samples with one order lower if the data for high and low frequency were ignored.

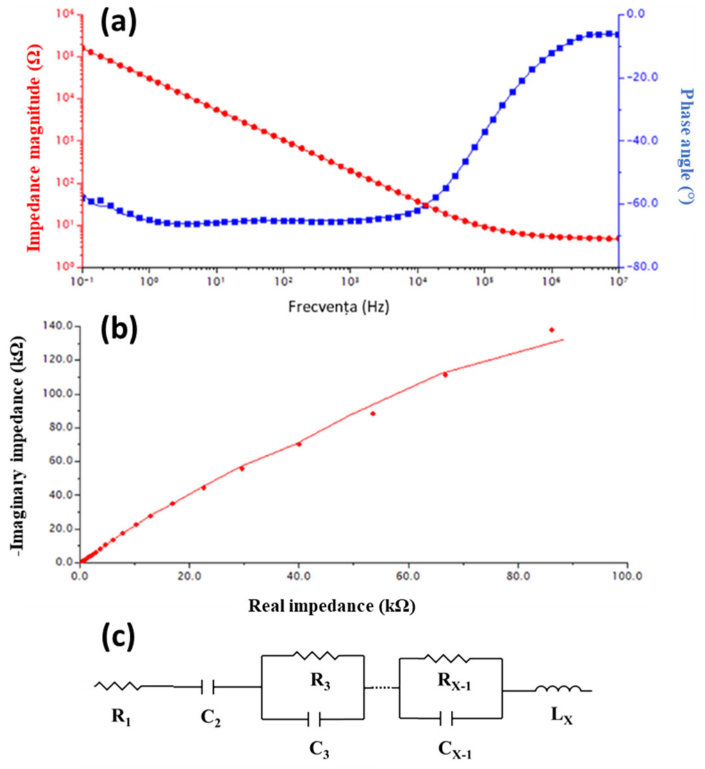

Figure S3. The Kramers-Kroning test for the data set of SPEEK membrane at 20°C: (a) Bode plot; (b) Nyquist plot; (c) the equivalent circuit used [60].

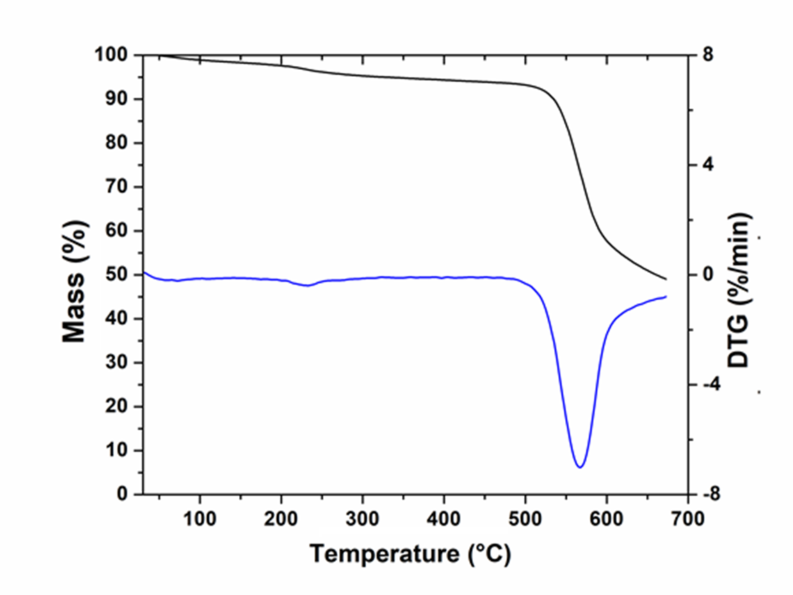

Figure S4. TG/DTG curves of PEEK.

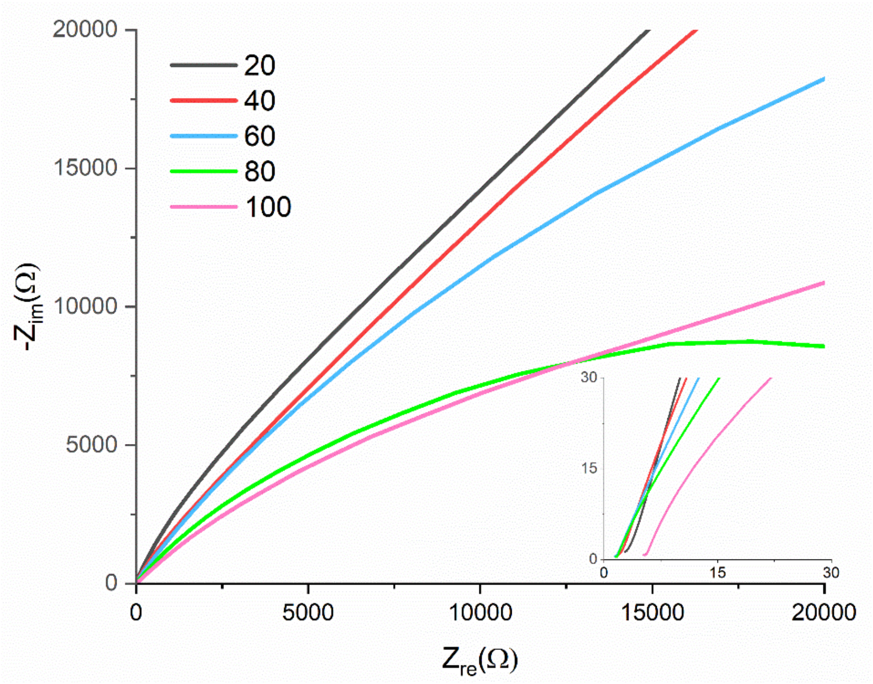

Figure S5. Nyquist plot for the SPEEK/Zn0.25 membrane at different temperatures.

Antioxidant activity

The antioxidant efficiency of praseodymium doped zinc ferrite was assessed by DPPH scavenging method. For this 2.7 mL of ethanolic DPPH solution of 20 mg/L was mixed 300 µL of doped zinc ferrite ethanol dispersion, the sample (100 µg/ml doped zinc ferrite) was placed at room temperature in the dark for 60 minutes. An UV-VIS spectrophotometer was used to measure the absorbance (A) at 517 nm wavelength, and the antioxidant activity was calculated with the following relation:

$$\text{Antioxidant activity (\%)} = \frac{A_{\text{control}} - A_{\text{sample}}}{A_{\text{control}}} \times 100 \quad \textbf{(3)}$$

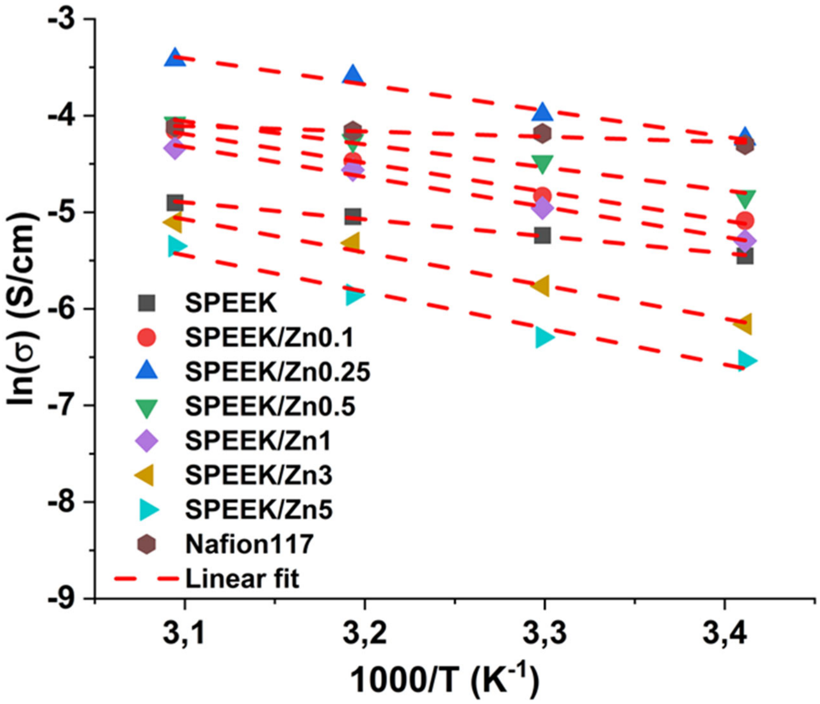

Figure S6. The Arrhenius plot of conductivity as a function of temperature for SPEEK and SPEEK/doped ferrite composite membranes

References

70. AfterMath Electrochemical Studio 1.6.10523 | Pine Research Instrumentation, (n.d.). https://pineresearch.com/downloads/aftermath-1-6-10523/.
